# Iron Excess Disturbs Metabolic Status and Relative Gonad Mass in Rats on High Fat, Fructose, and Salt Diets

**DOI:** 10.1007/s12011-012-9548-9

**Published:** 2012-11-22

**Authors:** Joanna Suliburska, Paweł Bogdański, Monika Szulińska

**Affiliations:** 1 Department of Human Nutrition and Hygiene, Poznan University of Life Sciences, Wojska Polskiego 31 Str., 60-624 Poznan, Poland; 2 Department of Internal Medicine, Metabolic Disorders and Hypertension, Poznan University of Medical Sciences, Poznan, Poland

**Keywords:** Iron, Fat, Fructose, Salt, Insulin, Nitric oxide, Zinc, Copper, Calcium

## Abstract

The aim of this study was to assess the metabolic and physiological changes in rats fed a diet high in fat, fructose, and salt, and with excess iron level. Mineral status was also estimated. Wistar rats were assigned to groups fed either a standard control diet (C) or a diet high in fat, fructose, and salt. The noncontrol diets contained either normal (M) or high level (MFe) of iron. After 6 weeks, the length and weight of the rats were measured, and the animals were euthanized. The kidneys and gonads were collected, and blood samples were taken. Serum levels of insulin, nitric oxide, and iron were measured. The iron, zinc, copper, and calcium concentrations of tissues were determined. It was found that the M diet led to a significant increase in the relative kidney mass of the rats compared with the control group. Among the rats fed the M diet, markedly higher serum level of iron and lower levels of zinc and copper were observed in tissues, while significantly higher calcium levels were found in the gonads. The MFe diet resulted in decreased obesity index, insulin level, and nitric oxide serum concentration in the rats, when compared with both the M and C diets. The high iron level in the modified diet increased the relative mass of the gonads. The excess iron level in the diet disturbed the zinc, copper, and calcium status of tissues. The decrease in insulin and nitric oxide in rats fed the diet high in iron, fat, fructose, and salt was associated with disorders of zinc, copper, and calcium status, as well as with an increase in the relative mass of the gonads.

## Introduction

According to the WHO, iron deficiency is the commonest and most widespread nutritional disorder in developed and developing counties [1]. Iron-deficient people typically need to take iron supplements. Yet there are reports that there exists a tendency toward the increased consumption of dietary supplements in developed countries, and this may result in overdoses of certain nutrients, including iron [2]. The taking of dietary supplements is often connected with improper dietary habits, characterized by the high intake of fat, sugar, salt, red meat, and refined grains. It is known that a diet rich in fat, simple sugars, and sodium induces the development of obesity and cardiovascular diseases. The results obtained from several studies show that high-salt diets decrease the level of nitric oxide in the plasma, induce hypertension, increase oxidative stress, and influence kidney dysfunction. It has been observed that high-fat and high-fructose diets increase oxidative stress and reduce nitric oxide bioavailability in rats [3, 4]. It is interesting to note that an excess of iron in the body also causes similar metabolic disorders. An association has been shown to exist between iron overload and oxidative stress, insulin resistance, and apoptosis of pancreatic cells [5–7]. Some authors have found that increased levels of iron stored in the body, expressed as serum ferritin concentration, are an independent predictor of diabetes [8]. Iron overload is also associated with renal dysfunction and hypogonadism [9, 10]. It is known that iron interacts with minerals such as zinc, copper, and calcium, disturbing their status in the body. These minerals are involved in the proper metabolic function of the body, including the synthesis and activity of insulin and the regulation of blood pressure [11].

It is unclear whether the high level of iron in the high fat, fructose, and salt diet enhances metabolic changes in the body. The question of whether the adverse metabolic changes caused by high level of iron in the body and modified diet are in fact associated with changes in mineral status—including zinc, copper, and calcium—also seems to be important. The aims of this study were to assess insulin and nitric oxide levels in the serum and to estimate zinc, copper, and calcium concentrations in selected tissues in rats fed a diet high in fat, fructose, and salt and containing excess iron level.

## Material and Methods

### Animals

All animal procedures were performed according to approved protocols and in accordance with the recommendations for the proper care and use of laboratory animals. The experiment was performed with the agreement of the local bioethics committee (approval no. 44/2007).

Six-week-old male Wistar rats (with mean initial body weight of 187 ± 13.5 g) were obtained from the Department of Toxicology, University of Medical Sciences, Poznań. The animals were housed individually in stainless steel cages coated with metal-free enamel, and kept under cycles of 12-h light to 12-h dark. Room temperature was maintained at 21 ± 1 °C with 55–65 % humidity.

### Experimental Design

The experiment was performed using 30 male Wistar rats. The animals were randomly assigned to three groups equal in size: control (C), modified diet with normal iron level (M), and modified diet with high iron level (MFe). The control group was fed a semisynthetic AIN-93M diet, while the modified group was fed a semisynthetic diet high in fat, fructose, and salt. The full composition of the diets is presented in Table 1. The amount of iron in the diet (“normal” or “high”) is shown in Table 2. In the normal-iron diet, the recommended concentration of iron was added to the mineral mixes: 6.06 g ferric citrate/kg mix. The high-iron diet was prepared by mixing an appropriate proportion of the AIN-93M diet with ferric sulfate (Merck).

**Table 1:** Ingredient and nutrient composition of the diets (grams per kilogram diet)

Ingredient	Control (C)	Modified (M)
Casein	140	140
Wheat starch	625	–
Sucrose	100	–
Potato starch	50	–
Vitamin mixture^a^	10	10
Mineral mixture^b^	35	35
Sunflower oil	40	40
Lard	–	150
Sodium chloride	–	35
Fructose	–	590
Total energy (kcal/100 g diet)	424	510
Total protein (% of energy)	13	13
Total fat (% of energy)	4	18

Control, control diet, modified, high fat, fructose and salt diet, *n* = 10 rats/group

^a^Composition of vitamin mixture (grams per kilogram mix): nicotinic acid (3), Ca pantothenate (1.6), pyridoxine (0.7), thiamin (0.6), riboflavin (0.6), folic acid (0.2), biotin (0.02), vitamin B12 (0.003); vitamin E (500 IU/g), vitamin A (500,000 IU/g), vitamin D3 (400,000 IU/g), vitamin K1 (0.08), choline bitartrate (200), powdered sucrose (777.15)

^b^Composition of mineral mixture (grams per kilogram mix): calcium carbonate (357), potassium phosphate monobasic (250), potassium citrate (28), sodium chloride (74), potassium sulfate (46.6), magnesium oxide (24), ferric citrate (6.06),zinc carbonate (1.65), sodium meta-silicate⋅9H_2_0 (1.45), manganous carbonate (0.64), cupric carbonate (0.30), chromium chloride⋅6H_2_0 (0.147), boric acid (0.0816), sodium fluoride (0.0635), nickel chloride⋅6H_2_0 (0.0578), lithium sulfate⋅H_2_0 (0.0263), sodium selenate anhydrous (0.0103), potassium iodate (0.010), ammonium paramolybdate⋅4H_2_0 (0.0795), ammonium vanadate (0.066), powdered sucrose (209.758)

**Table 2:** Concentration of iron in normal and high-iron diets

	C	M	MFe
Fe (mg/kg diet)	35.1 ± 0.5	37.1 ± 0.6	368.2 ± 2.3

Values are presented as mean ± SD

*C* control diet, *M* modified and normal iron diet, *MFe* modified and high iron diet

All rats were provided ad libitum diet and distilled water for 42 days. Dietary intake was recorded daily. Body weight was recorded each week before food distribution.

### Tissue and Serum Collection

At the end of the experimental period, the animals were weighed and their length measured. After 12 h of fasting, the rats were anesthetized with a sodium thiopental injection (40 mg/kg body weight) and killed by cardiac puncture. Blood was collected. The kidneys and gonads were dissected, weighed, and stored frozen (−70 °C) until analysis for mineral contents. The blood samples were collected by cardiac puncture in serum-separated tubes (without using an anticoagulant). The coagulated blood was left to clot at room temperature for 30 min, and then centrifuged for 15 min at 2,000 rpm at 4 °C. The supernatant fluid was then separated. Serum samples were stored at −70 °C for analysis.

### Biochemical Measurements

Serum insulin was determined by the radioimmunoassay method, using a rat insulin RIA kit (Insulin RIA Kit; Linco Research, Inc., USA). The estimated intra-assay coefficient of variation was <5 %.

The concentration of nitric oxide (NO) in the serum was determined by the spectrophotometric method, using a testing set from Oxis. The test determines nitric oxide concentrations based on the enzymatic conversion of nitrate to nitrite by nitrate reductase. This reaction is followed by colorimetric detection of nitrate as an azo dye product of the Griess reaction. The Griess reaction transforms nitrites to NO, which then reacts with sulfanilic acid. The indirect product of this reaction interacts with *N*-(1-naphtyl) ethylendiamine, leading to the formation of a product, red-violet in color, having maximum absorbance at *λ* = 540 nm. The solution color change, measured spectrophotometrically (using a Hyperion Micro Reader), is then proportional to the nitric oxide concentration. The accuracy of the method was verified with a primary reference material (BD11, Nederlands Meetinstituut), and amounted to 93 % [12].

### Determination of Minerals

The iron, zinc, copper, and calcium content in the serum and tissues was determined after digestion in 65 % (*w*/*w*) spectra pure HNO_3_ (Merck) in the Microwave Digestion System (MARS 5, CEM Corp., USA). Thereafter, the concentrations of iron, zinc, copper, and calcium in the mineral solutions were measured using the flame atomic absorption spectrometry method (AAS-3, Carl Zeiss, Jena, Germany). The content of minerals in the serum and tissues was determined at the following wavelengths: for iron, 248.3 nm; for zinc, 213.9 nm; for copper, 324.8 nm; and for calcium, 422.7 nm. The accuracy of the method was verified with certified reference materials (pig kidney BCR no. 186, Brussels) and was 92 % for iron, 95 % for zinc, 102 % for copper, and 93 % for calcium.

### Statistical Analysis

Detailed statistical analysis was performed using Statistica for Windows 10.0 (StatSoft, Inc., Poland). The results were expressed as arithmetic means and standard errors. One-way analysis of variance, followed by a post hoc Turkey’s test and *t* test, was used to compare the data between groups. Significance was set at the *P* < 0.05 level.

## Results

### Diet and Iron Intake

The average intake of the diet was comparable among groups (Table 3). The average daily intake of iron was comparable between the control group (C) and the group with the modified normal-iron diet (M). The intake of iron in the group with the MFe diet was about ten times higher than in groups C and M (Table 3).

**Table 3:** Daily diet and iron intake in rats

	C	M	MFe
Diet (g/day/rat)	21.2 ± 1.2	18.9 ± 1.6	18.2 ± 0.5
Fe (mg/day/rat)	0.74 ± 0.01	0.71 ± 0.01	6.75 ± 0.03

Values are presented as mean ± SD, *n* = 10 rats/group

*C* control diet, *M* modified and normal iron diet, *MFe* modified and high iron diet

### Biochemical Parameters in Serum

It was found that insulin and nitric oxide concentrations in the serum of the rats in the C and M groups were comparable (Table 3). The modified diet with high iron level (MFe) resulted in a significant decrease in insulin and nitric oxide levels in serum. A markedly higher level of iron in the serum of rats fed the modified diet (M and MFe groups) was also observed (Table 3).

### Body and Tissue Weight

The initial body weights were comparable among groups, but final body weight was significantly lower in the MFe group than in the C and M groups (Table 4). The modified diet with the normal iron level did not lead to a marked change in the obesity index of the rats. The excess iron level in the modified diet significantly decreased the obesity index, as compared to the M and C groups (Table 4). The relative kidney masses in rats fed the modified diet with (MFe) and without (M) excess iron were significantly higher than in the C group (Table 4). The high iron, fat, fructose, and salt diet led to a marked increase in the relative mass of the gonads in rats, as compared to the C and M groups (Table 4).

**Table 4:** Body and tissue weight and biochemical parameters in rats

	C	M	MFe	*P*
Initial body weight (g)	187.8 ± 19.5	187.8 ± 13.7	187.7 ± 15.2	NS
Final body weight (g)	346.5 ± 19.7^b^	332.0 ± 22.8^b^	298.5 ± 12.0^a^	<0.05
Obesity index	307.6 ± 14.5^b^	303.9 ± 11.0^b^	280.4 ± 3.2^a^	<0.01
Relative kidney weight (% b.m.)	0.7 ± 0.02^a^	0.8 ± 0.04^b^	0.8 ± 0.06^b^	<0.05
Relative gonad weight (% b.m.)	1.1 ± 0.1^a^	1.0 ± 0.2^a^	1.3 ± 0.1^b^	<0.05
Insulin in serum (pmol/l)	33.0 ± 5.9^b^	35.2 ± 5.1^b^	12.9 ± 1.4^a^	<0.05
Nitric oxide in serum (μmol/l)	10.0 ± 2.5^b^	10.6 ± 2.8^b^	8.6 ± 2.4^a^	<0.05
Iron in serum (μmol/l)	26.5 ± 1.1^a^	31.0 ± 1.1^b^	31.9 ± 0.7^b^	<0.05

Obesity index was calculated by dividing the cubic root of the body weight (grams) by the nasoanal length (millimeters) × 10^4^.Values are presented as mean ± SD, *n* = 10 rats/group, *P* level of significance

*C* control diet, *M* modified and normal iron diet, *MFe* modified and high iron diet

^a,b^significant differences between groups

### Contents of Minerals in Tissues

The high iron, fat, fructose, and salt diet markedly increased iron level while decreasing calcium content in the kidneys. The modified diets (M and MFe) resulted in a significant decrease in zinc and copper in the kidneys and a marked increase in calcium concentration in the gonads (Table 5). The copper content in the gonads of rats in the MFe group was markedly lower than that found in the C and M groups (Table 5).

**Table 5:** Contents of minerals in kidneys and gonads of rats

	C	M	MFe	*P*
Kidneys				
Fe (μg/g d.w.)	169.6 ± 10.7^a^	156.3 ± 10.8^a^	220.6 ± 18.3^b^	<0.05
Zn (μg/g d.w.)	107.7 ± 13.8^b^	92.1 ± 6.4^a^	88.2 ±5.7^a^	<0.05
Cu (μg/g d.w.)	33.6 ± 2.6^c^	24.3 ± 4.6^b^	11.9 ± 1.0^a^	<0.05
Ca (μg/g d.w.)	163.9 ± 5.2^b^	166.8 ± 25.8^b^	113.7 ± 25.1^a^	<0.05
Gonads				
Fe (μg/g d.w.)	139.2 ± 11.8	122.6 ± 16.8	127.8 ± 8.5	NS
Zn (μg/g d.w.)	169.4 ± 12.3	156.3 ± 8.7	141.0 ± 10.0	NS
Cu (μg/g d.w.)	10.8 ± 0.8^b^	8.85 ± 1.0^b^	7.0 ± 0.9^a^	<0.05
Ca (μg/g d.w.)	110.8 ± 26.4^a^	195.8 ± 21.4^b^	166.3 ± 28.2^b^	<0.01

Values are presented as mean ± SD, *n* = 10 rats/group, *P* level of significance

*C* control diet, *M* modified and normal iron diet, *MFe* modified and high iron diet, *d.w.* dry weight

^a,b^significant differences between groups

## Discussion

The aim of using high fat, fructose, and salt diets in this study was to develop metabolic changes in the rats, including increases in blood pressure. However, it was found here that the modified diets had no significant influence on insulin or nitric oxide concentrations in serum, although they did lead to increases in kidney size. Increased renal weight may be due to hypertrophy or hyperplasia following high sodium intake. It is possible that the renal enlargement observed in the growing rats is a mechanism for adapting to the intake of large amounts of salt. It can be supposed that this compensatory mechanism protects against increases in blood pressure in the growing rat. Previous studies have shown that a high-salt diet increases renal weight, and it has been suggested that this may be due to increases in the number of cells, enlargement of existing cells, or increases in interstitial tissue, water, protein, or fat. It has also been found that sodium chloride may initiate renal growth through changes in the circulation of renal growth factors [13, 14].

The other components modified in the diets—fat and fructose—may also have had an impact on kidney size. Manitius et al. [14] found that high-fructose, high-salt diets increased kidney size in rats. They concluded that the mechanism underlying this increase in size was not known. In the same study, it was observed that excess fat and salt in the diet downregulated the renin-angiotensin-aldosterone system and led to an increase in blood pressure [14]. In some studies, it has been observed that fructose stimulates salt absorption in the intestine and kidneys, and so can impact the state of salt overload and hypertension [4]. Decreases in renal medullary endothelial nitric oxide synthase in fructose-fed rats have also been reported [15].

In this study, neither the concentration of nitric oxide nor the serum insulin level or obesity index was affected by the high fat, fructose, or salt diet. These results indicate that the modified diet had no effect on insulin metabolism, nitric oxide, or the development of obesity in rats within the 6 weeks of the experiment. Indeed, other studies suggest an association between excess fructose and sodium in the diet and the development of insulin resistance, endothelial dysfunction, and hypertension [4, 16, 17].

The lack of impact of the modified diets on insulin and nitric oxide levels in serum and on obesity index observed in this study could be caused by a too-short experimental period of 6 weeks. In recent studies, increased insulin resistance, and an increase in blood pressure, was observed in experiments on mice and rats lasting for 16 and 32 weeks [18, 19]. In short-term (4 weeks) human studies, high fructose diets have also not affected insulin sensitivity [20]. It has been observed that 6 weeks of high sodium diet can significantly increase blood pressure and insulin level in serum in rats, but the diets employed in those experiments contained more than twice the sodium used in this study [21].

In this study, the modified diet led to increased iron level in serum, but to decreased levels of zinc and copper in the kidneys of rats. The mineral disorders observed in the rats fed the modified diet may be caused by the high levels of fat and fructose in the diet, and also by the interactions between minerals. It has been found that a high fructose diet impairs copper status and leads to iron overload [22]. It has been shown that increased fat in the diet increases iron absorption [23], but there are also contradictory reports [24]. It has been found that high-fructose diets induce copper deficiency, probably through impaired duodenum Ctr-1 expression, which leads to lower copper absorption [22, 25]. The lower zinc concentration in the kidneys of rats on the modified diet may be caused by interactions between iron and zinc [26]. The results of this study confirm the data obtained by Wapnir and Devas [25], who have also found that high-fructose, high-fat diets decrease copper and zinc levels in the kidneys of rats.

In this study, we observed an increase in calcium concentration in the gonads of rats fed the modified diet. In other studies, it has been found that high fructose and high sodium diets affect the absorption of calcium, disturb calcium homeostasis, and enhance vascular calcification [27–29]. Most likely, it is by means of some currently unknown mechanism that diets high in fat, fructose, and salt affect the accumulation of calcium in the testes, where this element plays a special role in cell growth and spermatid differentiation [30].

In this study, it was found that the modified diet did not have any influence on insulin, nitric oxide levels, or obesity index within 6 weeks, but that excess iron in the modified diet caused significant decreases in these metabolic parameters. These changes may have been caused by an increase in iron ion concentration in the tissues. Iron ions participate in the generation of free radicals, which may damage pancreatic cells and reduce insulin synthesis. Another factor among the rats fed the high-iron diet is the decreased synthesis of insulin in the pancreas, as a result of the low levels of zinc and copper in this tissue [31–33].

It is known that NO-deficient states are characterized by oxidative stress, inflammation, and endothelial dysfunction [34]. Oxidative stress inhibits NO production by impairing eNOS expression and its activity, and an increased level of free radicals is also associated with the degradation of NO [35]. Low level of nitric oxide in the body may also be conducive to disorders of sexual function in males [36].

In this study, decreases in insulin and NO concentration were associated with lower obesity index in those rats with excess iron in their modified diet. In other studies, it has been observed that iron levels in the body are inversely correlated with BMI values, and it has also been found that iron affects the expression of genes responsible for fat metabolism [37, 38].

An increase in the relative weight of testes in the rats fed an excess of iron in the modified diet was observed in this study. This was probably due to the lower weight gain of these rats coupled with normal growth of the testicular tissues. This may be connected with the maintenance of normal reproductive function, despite the presence of other metabolic function disorders in this group of growing rats. The increase in calcium concentration may indicate a sharp increase in the testicular tissue of MFe rat testes, compared with the control group [39]. However, the observed decrease in copper level in the testicular tissue may have led to abnormal tissue structure and affected the quality of the sperm [40].

## Conclusion

The influence of excess iron in a diet high in fat, fructose, and salt on metabolic changes—including decreases in obesity index, insulin, and nitric oxide serum concentrations, as well as increases in the relative mass of gonads—is a novel finding of this study. The observed metabolic and physiological changes were associated with disorders of zinc, copper, and calcium status in the body. In this study, the high fat, fructose, and salt diet employed over the 6 weeks of the experiment did not affect body mass, insulin, or nitric oxide serum levels, but did lead to increased kidney mass and iron serum level, as well as to disorders in mineral status in the tissues of rats.
